# Monoclinic modification of 1,2-bis­(diphenyl­seleno­phosphino­yl)ethane

**DOI:** 10.1107/S1600536808035010

**Published:** 2008-10-31

**Authors:** Ali Nemati Kharat, Maryam Ahmadian, Ghasem Bakhoda, Alireza Abbasi

**Affiliations:** a School of Chemistry, University College of Science, University of Tehran, Tehran, Iran

## Abstract

The complete mol­ecule of the title compound, C_26_H_24_P_2_Se_2_, is generated by crystallographic 2-fold symmetry, with the rotation axis bisecting the central C—C bond.   The dihedral angle between the terminal aromatic rings is 74.1 (1)°.

## Related literature

For the synthesis and related compounds, see: Lobana (1992 ▶); Lobana *et al.* (2007 ▶). For the triclinic modification, whose mol­ecule lies on a center-of-inversion, see: Risto *et al.* (2007 ▶).
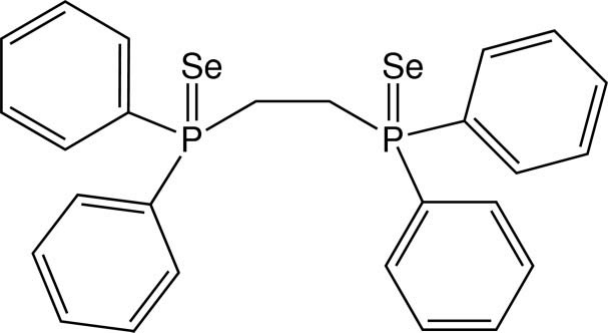


## Experimental

### 

#### Crystal data

C_26_H_24_P_2_Se_2_
*M* *_r_* = 556.31
Monoclinic, 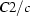

*a* = 15.828 (2) Å
*b* = 9.2057 (19) Å
*c* = 19.697 (3) Å
β = 121.654 (8)°
*V* = 2443.0 (7) Å^3^
*Z* = 4
Mo *K*α radiation
μ = 3.17 mm^−1^
*T* = 295 (2) K
0.20 × 0.12 × 0.11 mm

#### Data collection

Stoe IPDS-II diffractometer
Absorption correction: numerical (*X-SHAPE*; Stoe & Cie, 2007 ▶) *T*_min_ = 0.525, *T*_max_ = 0.701
8656 measured reflections
2361 independent reflections
1835 reflections with *I* > 2σ(*I*)
*R*_int_ = 0.072

#### Refinement

*R*[*F* ^2^ > 2σ(*F* ^2^)] = 0.052
*wR*(*F* ^2^) = 0.068
*S* = 1.15
2361 reflections
136 parameters
H-atom parameters constrained
Δρ_max_ = 0.49 e Å^−3^
Δρ_min_ = −0.41 e Å^−3^

### 

Data collection: *X-AREA* (Stoe & Cie, 2007 ▶); cell refinement: *X-AREA*; data reduction: *X-RED*; program(s) used to solve structure: *SHELXS97* (Sheldrick, 2008 ▶); program(s) used to refine structure: *SHELXL97* (Sheldrick, 2008 ▶); molecular graphics: *DIAMOND* (Brandenburg, 2001 ▶); software used to prepare material for publication: *PLATON* (Spek, 2003 ▶).

## Supplementary Material

Crystal structure: contains datablocks I, global. DOI: 10.1107/S1600536808035010/ng2502sup1.cif

Structure factors: contains datablocks I. DOI: 10.1107/S1600536808035010/ng2502Isup2.hkl

Additional supplementary materials:  crystallographic information; 3D view; checkCIF report

## Figures and Tables

**Table 1:** Selected bond lengths (Å)

P1—Se1	2.0979 (9)
P1—C1	1.822 (3)
P1—C2	1.809 (3)
P1—C8	1.812 (3)

**Fig. 1.**
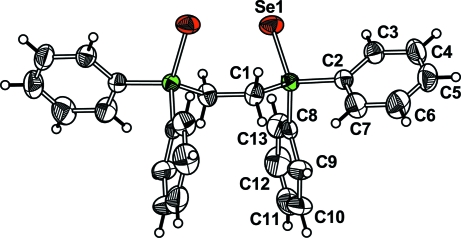
Molecular structure of (I), with 50% probability displacement ellipsoids. H atoms are shown as circles of arbitrary radii.

## supplementary crystallographic information

### Comment

Organophosphorous derivatives are an important class of ligands due to their potentially applications as selective homogenous catalysis. Among this group, the increasing interest in diphosphines and their calcogenide derivatives arises from their interesting coordination properties, extractive metallurgy and catalytic properties (Lobana, 1992; Lobana, *et al.* 2007). Therefore, we prompted to synthesis a new derivative of calcogenide organophosphorous ligands in order to investigate its structure.

The molecular structure of (I) and the atom-numbering scheme are shown in Fig. 1. The title compound was previously published in triclinic crystal system with two half-molecules in the asymmetric unit, having inversion symmetry for both molecules (Risto, *et al.* 2007). Here we presents the structure in monoclinic system in which, the asymmetric unit is one half-molecule. Two Diphenylphosphinoselenoyl, (PPh2)Se, are connected through an ethane group. Two phenyl rings (C2—C7 & C8—C13) in the asymmetric are located with dihedral angle of 74.1 (1)*^o^*. Weak inter-molecular hydrogen bonds (C10—H10···Se1^ii^, 3.751 (4) Å) are present between neighboring molecules, assembling the molecules into a three dimensional network.

### Experimental

Diphenylphosphinoethane (dppe) was prepared according to literature (Lobana, 1992). To a mixture of 3.98 g (0.01 mol) dppe in 300 ml of dried chloroform was added 1.58 g (0.02 mol) of red selenium. The reaction mixture was refluxed overnight and filtered the unreacted Se out. The resulting solution was evaporated under reduced pressure. The crystals suitable for crystallography were obtained by recrystallization from chloroform-acetonitrile (1:1).

### Refinement

All H atoms were placed in calculated positions and constrained to ride on their parent atoms (*U*_iso_(H) = 1.2(C)), with C—H = 0.93 and 0.97 Å, for aromatic and methylene, respectively.

### Crystal data

**Table:**

C_26_H_24_P_2_Se_2_	*F*(000) = 1112
*M**_r_* = 556.31	*D*_x_ = 1.513 Mg m^−^^3^
Monoclinic, *C*2/*c*	Mo *K*α radiation, λ = 0.71073 Å
Hall symbol: -C 2yc	Cell parameters from 8656 reflections
*a* = 15.828 (2) Å	θ = 2.5–29.5°
*b* = 9.2057 (19) Å	µ = 3.17 mm^−^^1^
*c* = 19.697 (3) Å	*T* = 295 K
β = 121.654 (8)°	Rod, colorless
*V* = 2443.0 (7) Å^3^	0.20 × 0.12 × 0.11 mm
*Z* = 4	

### Data collection

**Table:**

Stoe IPDS-II diffractometer	2361 independent reflections
Radiation source: fine-focus sealed tube	1835 reflections with *I* > 2σ(*I*)
graphite	*R*_int_ = 0.072
φ oscillation scans	θ_max_ = 26.0°, θ_min_ = 2.4°
Absorption correction: numerical (XSHAPE; Stoe & Cie, 2007)	*h* = −19→16
*T*_min_ = 0.525, *T*_max_ = 0.701	*k* = −11→11
8656 measured reflections	*l* = −23→24

### Refinement

**Table:**

Refinement on *F*^2^	Primary atom site location: structure-invariant direct methods
Least-squares matrix: full	Secondary atom site location: difference Fourier map
*R*[*F*^2^ > 2σ(*F*^2^)] = 0.052	Hydrogen site location: inferred from neighbouring sites
*wR*(*F*^2^) = 0.068	H-atom parameters constrained
*S* = 1.15	*w* = 1/[σ^2^(*F*_o_^2^) + (0.0166*P*)^2^ + 3.9345*P*] where *P* = (*F*_o_^2^ + 2*F*_c_^2^)/3
2361 reflections	(Δ/σ)_max_ = 0.001
136 parameters	Δρ_max_ = 0.49 e Å^−^^3^
0 restraints	Δρ_min_ = −0.41 e Å^−^^3^

### Special details

**Table:**

Geometry. All e.s.d.'s (except the e.s.d. in the dihedral angle between two l.s. planes) are estimated using the full covariance matrix. The cell e.s.d.'s are taken into account individually in the estimation of e.s.d.'s in distances, angles and torsion angles; correlations between e.s.d.'s in cell parameters are only used when they are defined by crystal symmetry. An approximate (isotropic) treatment of cell e.s.d.'s is used for estimating e.s.d.'s involving l.s. planes.
Refinement. Refinement of *F*^2^ against ALL reflections. The weighted *R*-factor *wR* and goodness of fit *S* are based on *F*^2^, conventional *R*-factors *R* are based on *F*, with *F* set to zero for negative *F*^2^. The threshold expression of *F*^2^ > σ(*F*^2^) is used only for calculating *R*-factors(gt) *etc*. and is not relevant to the choice of reflections for refinement. *R*-factors based on *F*^2^ are statistically about twice as large as those based on *F*, and *R*- factors based on ALL data will be even larger.

### Fractional atomic coordinates and isotropic or equivalent isotropic displacement parameters (Å^2^)

**Table:**

	*x*	*y*	*z*	*U*_iso_*/*U*_eq_	
Se1	0.44808 (3)	1.02455 (3)	0.10565 (2)	0.04679 (13)	
P1	0.52862 (6)	0.83149 (8)	0.15258 (5)	0.02995 (18)	
C1	0.5482 (2)	0.7912 (4)	0.25038 (19)	0.0396 (7)	
H1A	0.5795	0.6968	0.2678	0.048*	
H1B	0.5928	0.8630	0.2884	0.048*	
C2	0.6516 (2)	0.8360 (3)	0.16755 (18)	0.0333 (7)	
C3	0.6699 (2)	0.9256 (3)	0.1207 (2)	0.0427 (8)	
H3	0.6202	0.9872	0.0843	0.051*	
C4	0.7609 (3)	0.9253 (4)	0.1271 (2)	0.0563 (10)	
H4	0.7720	0.9856	0.0946	0.068*	
C5	0.8346 (3)	0.8363 (4)	0.1810 (3)	0.0600 (11)	
H5	0.8960	0.8361	0.1853	0.072*	
C6	0.8188 (3)	0.7477 (5)	0.2286 (3)	0.0661 (11)	
H6	0.8694	0.6873	0.2654	0.079*	
C7	0.7277 (3)	0.7473 (4)	0.2225 (2)	0.0536 (9)	
H7	0.7174	0.6871	0.2554	0.064*	
C8	0.4663 (2)	0.6748 (3)	0.09047 (19)	0.0350 (7)	
C9	0.5078 (3)	0.5379 (4)	0.1130 (2)	0.0547 (9)	
H9	0.5680	0.5255	0.1607	0.066*	
C10	0.4601 (4)	0.4197 (4)	0.0649 (3)	0.0745 (14)	
H10	0.4887	0.3280	0.0802	0.089*	
C11	0.3726 (4)	0.4361 (5)	−0.0039 (4)	0.0806 (16)	
H11	0.3407	0.3554	−0.0356	0.097*	
C12	0.3304 (3)	0.5698 (5)	−0.0274 (3)	0.0758 (13)	
H12	0.2699	0.5806	−0.0751	0.091*	
C13	0.3777 (3)	0.6897 (4)	0.0197 (2)	0.0532 (9)	
H13	0.3492	0.7812	0.0033	0.064*	

### Atomic displacement parameters (Å^2^)

**Table:**

	*U*^11^	*U*^22^	*U*^33^	*U*^12^	*U*^13^	*U*^23^
Se1	0.0441 (2)	0.03445 (18)	0.0531 (2)	0.00738 (15)	0.01948 (17)	0.00760 (16)
P1	0.0314 (4)	0.0303 (4)	0.0278 (4)	0.0010 (3)	0.0153 (3)	0.0020 (3)
C1	0.0389 (18)	0.0512 (19)	0.0291 (16)	0.0072 (14)	0.0181 (15)	0.0059 (14)
C2	0.0333 (17)	0.0365 (16)	0.0297 (16)	−0.0014 (13)	0.0161 (14)	−0.0020 (13)
C3	0.042 (2)	0.0475 (19)	0.0397 (19)	0.0000 (15)	0.0220 (17)	0.0029 (15)
C4	0.060 (3)	0.064 (2)	0.062 (3)	−0.0087 (19)	0.043 (2)	0.0009 (19)
C5	0.040 (2)	0.077 (3)	0.070 (3)	−0.006 (2)	0.033 (2)	−0.009 (2)
C6	0.038 (2)	0.079 (3)	0.072 (3)	0.017 (2)	0.023 (2)	0.014 (2)
C7	0.042 (2)	0.063 (2)	0.055 (2)	0.0097 (17)	0.0244 (19)	0.0205 (19)
C8	0.0389 (19)	0.0366 (16)	0.0392 (19)	−0.0057 (13)	0.0272 (17)	−0.0034 (14)
C9	0.069 (3)	0.0364 (18)	0.065 (3)	−0.0034 (17)	0.040 (2)	0.0005 (18)
C10	0.113 (4)	0.034 (2)	0.111 (4)	−0.016 (2)	0.083 (4)	−0.014 (2)
C11	0.096 (4)	0.073 (3)	0.108 (4)	−0.049 (3)	0.078 (4)	−0.053 (3)
C12	0.058 (3)	0.094 (3)	0.072 (3)	−0.028 (2)	0.031 (2)	−0.042 (3)
C13	0.045 (2)	0.060 (2)	0.049 (2)	−0.0064 (17)	0.021 (2)	−0.0139 (18)

### Geometric parameters (Å, °)

**Table:**

Se1—P1	2.0979 (9)	C6—C7	1.383 (5)
P1—C1	1.822 (3)	C6—H6	0.9300
P1—C2	1.809 (3)	C7—H7	0.9300
P1—C8	1.812 (3)	C8—C13	1.369 (5)
C1—C1^i^	1.517 (6)	C8—C9	1.382 (4)
C1—H1A	0.9700	C9—C10	1.378 (6)
C1—H1B	0.9700	C9—H9	0.9300
C2—C3	1.377 (4)	C10—C11	1.344 (7)
C2—C7	1.386 (5)	C10—H10	0.9300
C3—C4	1.379 (5)	C11—C12	1.361 (7)
C3—H3	0.9300	C11—H11	0.9300
C4—C5	1.364 (5)	C12—C13	1.383 (5)
C4—H4	0.9300	C12—H12	0.9300
C5—C6	1.362 (6)	C13—H13	0.9300
C5—H5	0.9300		
			
C2—P1—C8	106.64 (13)	C5—C6—C7	120.1 (4)
C2—P1—C1	105.13 (14)	C5—C6—H6	120.0
C8—P1—C1	106.37 (15)	C7—C6—H6	120.0
C2—P1—Se1	113.91 (10)	C6—C7—C2	120.4 (3)
C8—P1—Se1	112.79 (11)	C6—C7—H7	119.8
C1—P1—Se1	111.41 (11)	C2—C7—H7	119.8
C1^i^—C1—P1	112.2 (3)	C13—C8—C9	118.6 (3)
C1^i^—C1—H1A	109.2	C13—C8—P1	120.7 (2)
P1—C1—H1A	109.2	C9—C8—P1	120.7 (3)
C1^i^—C1—H1B	109.2	C10—C9—C8	120.2 (4)
P1—C1—H1B	109.2	C10—C9—H9	119.9
H1A—C1—H1B	107.9	C8—C9—H9	119.9
C3—C2—C7	118.2 (3)	C11—C10—C9	120.5 (4)
C3—C2—P1	119.4 (2)	C11—C10—H10	119.8
C7—C2—P1	122.3 (2)	C9—C10—H10	119.8
C2—C3—C4	121.1 (3)	C10—C11—C12	120.4 (4)
C2—C3—H3	119.5	C10—C11—H11	119.8
C4—C3—H3	119.5	C12—C11—H11	119.8
C5—C4—C3	119.8 (3)	C11—C12—C13	119.8 (4)
C5—C4—H4	120.1	C11—C12—H12	120.1
C3—C4—H4	120.1	C13—C12—H12	120.1
C6—C5—C4	120.3 (3)	C8—C13—C12	120.5 (4)
C6—C5—H5	119.8	C8—C13—H13	119.7
C4—C5—H5	119.8	C12—C13—H13	119.7
			
C2—P1—C1—C1^i^	176.96 (11)	P1—C2—C7—C6	176.0 (3)
C8—P1—C1—C1^i^	−70.17 (14)	C2—P1—C8—C13	−123.7 (3)
Se1—P1—C1—C1^i^	53.12 (12)	C1—P1—C8—C13	124.5 (3)
C8—P1—C2—C3	97.9 (3)	Se1—P1—C8—C13	2.1 (3)
C1—P1—C2—C3	−149.4 (3)	C2—P1—C8—C9	55.5 (3)
Se1—P1—C2—C3	−27.1 (3)	C1—P1—C8—C9	−56.3 (3)
C8—P1—C2—C7	−79.1 (3)	Se1—P1—C8—C9	−178.7 (2)
C1—P1—C2—C7	33.6 (3)	C13—C8—C9—C10	−0.2 (5)
Se1—P1—C2—C7	155.8 (3)	P1—C8—C9—C10	−179.4 (3)
C7—C2—C3—C4	1.2 (5)	C8—C9—C10—C11	−0.6 (6)
P1—C2—C3—C4	−176.0 (3)	C9—C10—C11—C12	0.8 (7)
C2—C3—C4—C5	−0.7 (6)	C10—C11—C12—C13	−0.1 (7)
C3—C4—C5—C6	0.0 (6)	C9—C8—C13—C12	0.8 (5)
C4—C5—C6—C7	0.1 (7)	P1—C8—C13—C12	−180.0 (3)
C5—C6—C7—C2	0.5 (6)	C11—C12—C13—C8	−0.7 (6)
C3—C2—C7—C6	−1.1 (5)		

Symmetry codes: (i) −*x*+1, *y*, −*z*+1/2.

### Hydrogen-bond geometry (Å, °)

**Table:**

*D*—H···*A*	*D*—H	H···*A*	*D*···*A*	*D*—H···*A*
C10—H10···Se1^ii^	0.93	2.97	3.751 (4)	143
C1—H1B···Se1^i^	0.97	2.90	3.533 (3)	124
C13—H13···Se1	0.93	2.87	3.410 (4)	119

Symmetry codes: (ii) *x*, *y*−1, *z*; (i) −*x*+1, *y*, −*z*+1/2.
